# (6,6-Dimethyl-1-phenyl-6,7-di­hydro-5*H*-pyrrolizin-2-yl)(thio­phen-2-yl)methanone

**DOI:** 10.1107/S1600536813023489

**Published:** 2013-09-07

**Authors:** Yu-min Liu, Jia-liang Zhong, Wen-xia Sun, Fu-li Zhang, He Liu

**Affiliations:** a College of Chemical and Pharmaceutical Engineering, Hebei University of Science and Technology, Shijiazhuan 050016, People’s Republic of China; b Shanghai Institute of Pharmaceutical Industry, Shanghai 200040, People’s Republic of China; c Beijing Chao-Yang Hospital Affiliated with Beijing Capital Medical University, Beijing 100020, People’s Republic of China

## Abstract

In the title compound, C_20_H_19_NOS, the pyrrolizine ring is essentially planar (r.m.s. deviation = 0.001 Å) while the fused dihydro-pyrrolizine ring adopts an envelope comformation with the C atom bearing the methyl substituents as the flap. The dihedral angles between the pyrrolizine and the phenyl and thio­phene rings are 34.54 (7) and 44.93 (7)°, respectively. In the crystal, weak C—H⋯O hydrogen bonds link the mol­ecules into infinite zigzag chains parallel to the *b*-axis direction.

## Related literature

For the synthesis of the title compound, see: Dannhardt & Obergrusberger (1979 ▶). For a similar structure, see: Liu *et al.* (2007 ▶).
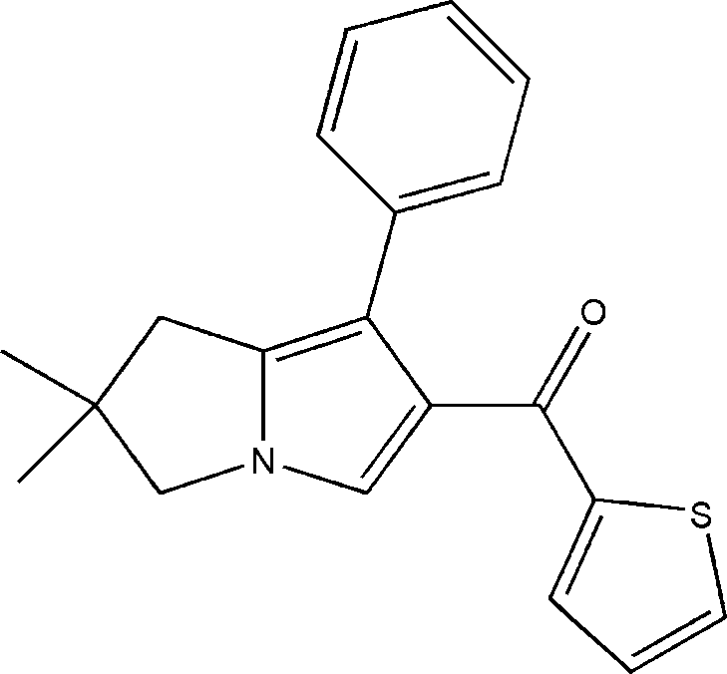


## Experimental

### 

#### Crystal data

C_20_H_19_NOS
*M* *_r_* = 321.42
Orthorhombic, 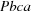

*a* = 16.251 (3) Å
*b* = 10.473 (2) Å
*c* = 19.973 (4) Å
*V* = 3399.3 (11) Å^3^
*Z* = 8
Mo *K*α radiation
μ = 0.19 mm^−1^
*T* = 296 K
0.22 × 0.19 × 0.12 mm

#### Data collection

Bruker APEXII CCD diffractometer
30107 measured reflections
3894 independent reflections
2743 reflections with *I* > 2σ(*I*)
*R*_int_ = 0.070

#### Refinement

*R*[*F* ^2^ > 2σ(*F* ^2^)] = 0.046
*wR*(*F* ^2^) = 0.131
*S* = 1.03
3894 reflections
209 parameters
H-atom parameters constrained
Δρ_max_ = 0.20 e Å^−3^
Δρ_min_ = −0.25 e Å^−3^

### 

Data collection: *APEX2* (Bruker, 2009 ▶); cell refinement: *SAINT* (Bruker, 2009 ▶); data reduction: *SAINT*; program(s) used to solve structure: *SHELXS97* (Sheldrick, 2008 ▶); program(s) used to refine structure: *SHELXL97* (Sheldrick, 2008 ▶); molecular graphics: *SHELXTL* (Sheldrick, 2008 ▶); software used to prepare material for publication: *SHELXTL*.

## Supplementary Material

Crystal structure: contains datablock(s) I, global. DOI: 10.1107/S1600536813023489/pk2493sup1.cif

Structure factors: contains datablock(s) I. DOI: 10.1107/S1600536813023489/pk2493Isup2.hkl

Supplementary material file. DOI: 10.1107/S1600536813023489/pk2493Isup3.cml

Additional supplementary materials:  crystallographic information; 3D view; checkCIF report

## Figures and Tables

**Table 1:** Hydrogen-bond geometry (Å, °)

*D*—H⋯*A*	*D*—H	H⋯*A*	*D*⋯*A*	*D*—H⋯*A*
C13—H13*A*⋯O1^i^	0.93	2.69	3.529 (3)	150

Symmetry code: (i) 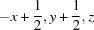.

**Fig. 1.**
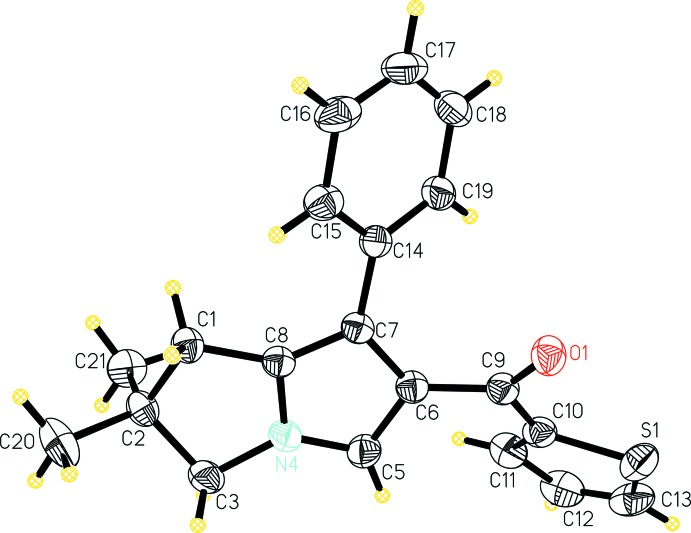
View of the molecule of (I) showing displacement ellipsoids drawn at the 30% probability level.

## supplementary crystallographic information

### 1. Comment

The title compound, (I) (Fig. 1), 6-(2-thenoyl-)-7-phenyl-2,2-dimethyl- 2,3-dihydro-1*H*-pyrrolizine was synthesized from 5-phenyl- 3,3-dimethyl-3,4-dihydro-1*H*-pyrrolizine and 3-bromo-2-thenoylethanone according to a general literature of Dannhardt *et al.* (1979).

Compared with the structure of C_20_H_17_ON (Liu *et al.*, 2007), the thenoyl group results in a larger calculated density for the crystal. The 2,3-dihydro-1*H*-pyrrolizine ring A (C1/C2/C3/N4 /C5/C6/C7/C8) adopts an almost planar conformation except the carbon atom which links to the methyl groups, with the mean deviation from the least-squares plane being 0.1064 Å, The dihedral angle between the pyrrolizine and phenyl rings (C14—C19) is 34.54 (7)°, the dihedral angle between pyrrolizine and thiophene rings (C10/C11/C12/C13/S1) is 44.93 (7)°, and the dihedral angle between phenyl and thiophene rings is 68.72 (6)°.

In the crystal, weak intermolecular C—H···O (Table 1) hydrogen bonds can be found, linking adjacent molecules along the *b* axis to form one-dimensional zigzag chains, which contributes to the stable packing of molecules in the crystal. No π-π stacking interactions were found in this crystal structure.

### 2. Experimental

A stirred solution of 5-phenyl-3,3-dimethyl-3,4-dihydro-1*H*-pyrrolizine in CH_2_Cl_2_ was treated with a solution of 3-bromo-2-thenoylethanone. The mixture was stirred for 4 h at room temperature, an aqueous solution of NaHCO_3_ was added and stirred for 3 h. Then water was added to form a clear aqueous layer, the organic layer was separated and dried (anhydrous Na_2_SO_4_) before the solvent was evaporated. The solution was evaporated under reduced pressure and purified by chromatography on a silica gel column, eluting with a petroleum ether/acetone mixture to give 32% yield of light yellow solid. The purity of the title compound was verified by elemental analysis: calculated for C_20_H_19_NOS: C 74.73, H 5.96, N 4.36; found C 74.59, H 5.98, N 4.35. EI—MS m/*z*: 322(*M*+H)^+^.

The crystal appropriate for X-ray data collection was obtained from DMF-H_2_O solution at room temperature after about a week.

### 3. Refinement

All H atoms were placed in geometically idealized positions and constrained to ride on their parent atoms with C—H distances of 0.93 (0.97 for CH_2_)Å for CH, and *U*_iso_(H) = 1.2 (1.5 for CH_3_) *U*_eq_(C).

### Crystal data

**Table:**

C_20_H_19_NOS	*F*(000) = 1360
*M**_r_* = 321.42	*D*_x_ = 1.256 Mg m^−^^3^
Orthorhombic, *P**b**c**a*	Mo *K*α radiation, λ = 0.71073 Å
Hall symbol: -P 2ac 2ab	Cell parameters from 17698 reflections
*a* = 16.251 (3) Å	θ = 3.1–27.5°
*b* = 10.473 (2) Å	µ = 0.19 mm^−^^1^
*c* = 19.973 (4) Å	*T* = 296 K
*V* = 3399.3 (11) Å^3^	Prismatic, colorless
*Z* = 8	0.22 × 0.19 × 0.12 mm

### Data collection

**Table:**

Bruker APEXII CCD diffractometer	2743 reflections with *I* > 2σ(*I*)
Radiation source: fine-focus sealed tube	*R*_int_ = 0.070
Graphite monochromator	θ_max_ = 27.5°, θ_min_ = 3.1°
φ and ω scans	*h* = −21→20
30107 measured reflections	*k* = −13→13
3894 independent reflections	*l* = −25→25

### Refinement

**Table:**

Refinement on *F*^2^	Secondary atom site location: difference Fourier map
Least-squares matrix: full	Hydrogen site location: inferred from neighbouring sites
*R*[*F*^2^ > 2σ(*F*^2^)] = 0.046	H-atom parameters constrained
*wR*(*F*^2^) = 0.131	*w* = 1/[σ^2^(*F*_o_^2^) + (0.0601*P*)^2^ + 0.6291*P*] where *P* = (*F*_o_^2^ + 2*F*_c_^2^)/3
*S* = 1.03	(Δ/σ)_max_ = 0.001
3894 reflections	Δρ_max_ = 0.20 e Å^−^^3^
209 parameters	Δρ_min_ = −0.25 e Å^−^^3^
0 restraints	Extinction correction: *SHELXL97* (Sheldrick, 2008), Fc^*^=kFc[1+0.001xFc^2^λ^3^/sin(2θ)]^-1/4^
Primary atom site location: structure-invariant direct methods	Extinction coefficient: 0.0049 (11)

### Special details

**Table:**

Geometry. All e.s.d.'s (except the e.s.d. in the dihedral angle between two l.s. planes) are estimated using the full covariance matrix. The cell e.s.d.'s are taken into account individually in the estimation of e.s.d.'s in distances, angles and torsion angles; correlations between e.s.d.'s in cell parameters are only used when they are defined by crystal symmetry. An approximate (isotropic) treatment of cell e.s.d.'s is used for estimating e.s.d.'s involving l.s. planes.
Refinement. Refinement of *F*^2^ against ALL reflections. The weighted *R*-factor *wR* and goodness of fit *S* are based on *F*^2^, conventional *R*-factors *R* are based on *F*, with *F* set to zero for negative *F*^2^. The threshold expression of *F*^2^ > σ(*F*^2^) is used only for calculating *R*-factors(gt) *etc*. and is not relevant to the choice of reflections for refinement. *R*-factors based on *F*^2^ are statistically about twice as large as those based on *F*, and *R*- factors based on ALL data will be even larger.

### Fractional atomic coordinates and isotropic or equivalent isotropic displacement parameters (Å^2^)

**Table:**

	*x*	*y*	*z*	*U*_iso_*/*U*_eq_	
S1	0.20601 (3)	0.94554 (5)	0.16441 (3)	0.0689 (2)	
N4	0.05241 (9)	0.72368 (14)	−0.07701 (7)	0.0509 (4)	
O1	0.06950 (8)	0.76239 (12)	0.14671 (6)	0.0612 (4)	
C1	−0.06376 (11)	0.60225 (16)	−0.10279 (8)	0.0492 (4)	
H1A	−0.1219	0.6232	−0.1019	0.059*	
H1B	−0.0573	0.5108	−0.0971	0.059*	
C2	−0.02331 (12)	0.64795 (16)	−0.16906 (8)	0.0494 (4)	
C3	0.06294 (12)	0.6923 (2)	−0.14784 (9)	0.0597 (5)	
H3A	0.1031	0.6247	−0.1537	0.072*	
H3B	0.0801	0.7665	−0.1733	0.072*	
C5	0.09527 (12)	0.78755 (17)	−0.02986 (9)	0.0542 (4)	
H5A	0.1451	0.8294	−0.0361	0.065*	
C6	0.05165 (10)	0.77953 (16)	0.02961 (8)	0.0471 (4)	
C7	−0.02153 (10)	0.70597 (15)	0.01645 (8)	0.0434 (4)	
C8	−0.01806 (10)	0.67366 (15)	−0.05008 (8)	0.0442 (4)	
C9	0.08533 (11)	0.81958 (16)	0.09447 (9)	0.0484 (4)	
C10	0.14245 (11)	0.92911 (17)	0.09620 (9)	0.0524 (4)	
C11	0.15343 (13)	1.02868 (18)	0.05123 (10)	0.0645 (5)	
H11A	0.1237	1.0383	0.0117	0.077*	
C12	0.21582 (15)	1.1140 (2)	0.07320 (14)	0.0796 (7)	
H12A	0.2320	1.1860	0.0493	0.096*	
C13	0.24891 (14)	1.0797 (2)	0.13224 (15)	0.0806 (7)	
H13A	0.2909	1.1248	0.1532	0.097*	
C14	−0.09238 (10)	0.67833 (16)	0.06018 (8)	0.0461 (4)	
C15	−0.13925 (11)	0.56785 (18)	0.04985 (9)	0.0569 (5)	
H15A	−0.1243	0.5113	0.0161	0.068*	
C16	−0.20727 (12)	0.5415 (2)	0.08900 (11)	0.0722 (6)	
H16A	−0.2377	0.4678	0.0813	0.087*	
C17	−0.22995 (12)	0.6230 (3)	0.13876 (11)	0.0808 (8)	
H17A	−0.2751	0.6039	0.1656	0.097*	
C18	−0.18603 (13)	0.7339 (3)	0.14950 (10)	0.0761 (7)	
H18A	−0.2025	0.7903	0.1829	0.091*	
C19	−0.11707 (11)	0.7617 (2)	0.11052 (9)	0.0592 (5)	
H19A	−0.0875	0.8363	0.1182	0.071*	
C20	−0.01924 (18)	0.5406 (2)	−0.22061 (11)	0.0838 (7)	
H20A	0.0061	0.5718	−0.2609	0.126*	
H20B	−0.0739	0.5114	−0.2305	0.126*	
H20C	0.0127	0.4712	−0.2030	0.126*	
C21	−0.07040 (13)	0.76133 (19)	−0.19715 (10)	0.0665 (5)	
H21A	−0.0450	0.7890	−0.2380	0.100*	
H21B	−0.0697	0.8299	−0.1653	0.100*	
H21C	−0.1263	0.7367	−0.2059	0.100*	

### Atomic displacement parameters (Å^2^)

**Table:**

	*U*^11^	*U*^22^	*U*^33^	*U*^12^	*U*^13^	*U*^23^
S1	0.0568 (3)	0.0595 (3)	0.0903 (4)	0.0037 (2)	−0.0164 (3)	−0.0125 (3)
N4	0.0503 (8)	0.0583 (9)	0.0442 (8)	−0.0047 (7)	0.0079 (6)	0.0023 (7)
O1	0.0671 (9)	0.0656 (8)	0.0510 (7)	−0.0096 (7)	−0.0037 (6)	0.0062 (6)
C1	0.0562 (10)	0.0472 (9)	0.0441 (9)	−0.0030 (8)	0.0003 (7)	0.0017 (7)
C2	0.0630 (11)	0.0439 (9)	0.0412 (9)	0.0014 (8)	0.0037 (8)	0.0021 (7)
C3	0.0573 (11)	0.0773 (13)	0.0446 (10)	0.0031 (10)	0.0102 (8)	0.0047 (9)
C5	0.0503 (10)	0.0589 (10)	0.0534 (10)	−0.0108 (8)	0.0017 (8)	0.0029 (8)
C6	0.0488 (9)	0.0461 (9)	0.0464 (9)	−0.0030 (7)	0.0019 (7)	0.0029 (7)
C7	0.0457 (9)	0.0418 (8)	0.0427 (8)	0.0019 (7)	0.0032 (7)	0.0047 (7)
C8	0.0457 (9)	0.0434 (8)	0.0436 (9)	−0.0001 (7)	0.0033 (7)	0.0055 (7)
C9	0.0477 (9)	0.0484 (9)	0.0491 (10)	0.0014 (8)	0.0009 (8)	−0.0011 (8)
C10	0.0477 (10)	0.0490 (9)	0.0605 (11)	0.0021 (8)	0.0036 (8)	−0.0086 (8)
C11	0.0735 (13)	0.0483 (10)	0.0716 (13)	−0.0088 (9)	0.0124 (10)	−0.0058 (9)
C12	0.0830 (16)	0.0533 (12)	0.1026 (18)	−0.0138 (11)	0.0246 (14)	−0.0115 (12)
C13	0.0539 (12)	0.0616 (13)	0.126 (2)	−0.0075 (10)	0.0059 (14)	−0.0283 (14)
C14	0.0432 (9)	0.0569 (10)	0.0382 (8)	0.0063 (8)	−0.0021 (7)	0.0100 (7)
C15	0.0527 (10)	0.0629 (11)	0.0552 (10)	−0.0034 (9)	0.0019 (8)	0.0143 (9)
C16	0.0492 (11)	0.0956 (16)	0.0717 (13)	−0.0099 (11)	0.0008 (10)	0.0301 (12)
C17	0.0427 (11)	0.139 (2)	0.0613 (13)	0.0102 (14)	0.0061 (9)	0.0335 (15)
C18	0.0584 (12)	0.126 (2)	0.0443 (11)	0.0307 (14)	0.0038 (9)	0.0037 (12)
C19	0.0518 (10)	0.0795 (13)	0.0463 (10)	0.0127 (10)	−0.0005 (8)	−0.0003 (9)
C20	0.130 (2)	0.0623 (13)	0.0588 (12)	−0.0102 (13)	0.0248 (13)	−0.0127 (10)
C21	0.0714 (13)	0.0656 (12)	0.0625 (12)	0.0036 (10)	−0.0002 (10)	0.0190 (10)

### Geometric parameters (Å, º)

**Table:**

S1—C13	1.695 (3)	C11—C12	1.421 (3)
S1—C10	1.7182 (19)	C11—H11A	0.9300
N4—C5	1.349 (2)	C12—C13	1.345 (3)
N4—C8	1.370 (2)	C12—H12A	0.9300
N4—C3	1.462 (2)	C13—H13A	0.9300
O1—C9	1.230 (2)	C14—C19	1.391 (3)
C1—C8	1.490 (2)	C14—C15	1.401 (3)
C1—C2	1.553 (2)	C15—C16	1.382 (3)
C1—H1A	0.9700	C15—H15A	0.9300
C1—H1B	0.9700	C16—C17	1.361 (3)
C2—C21	1.520 (2)	C16—H16A	0.9300
C2—C20	1.526 (2)	C17—C18	1.380 (4)
C2—C3	1.536 (3)	C17—H17A	0.9300
C3—H3A	0.9700	C18—C19	1.395 (3)
C3—H3B	0.9700	C18—H18A	0.9300
C5—C6	1.386 (2)	C19—H19A	0.9300
C5—H5A	0.9300	C20—H20A	0.9600
C6—C7	1.441 (2)	C20—H20B	0.9600
C6—C9	1.468 (2)	C20—H20C	0.9600
C7—C8	1.372 (2)	C21—H21A	0.9600
C7—C14	1.474 (2)	C21—H21B	0.9600
C9—C10	1.476 (2)	C21—H21C	0.9600
C10—C11	1.388 (3)		
			
C13—S1—C10	91.71 (12)	C10—C11—C12	111.4 (2)
C5—N4—C8	110.32 (14)	C10—C11—H11A	124.3
C5—N4—C3	136.60 (15)	C12—C11—H11A	124.3
C8—N4—C3	113.07 (14)	C13—C12—C11	112.8 (2)
C8—C1—C2	103.68 (14)	C13—C12—H12A	123.6
C8—C1—H1A	111.0	C11—C12—H12A	123.6
C2—C1—H1A	111.0	C12—C13—S1	112.92 (18)
C8—C1—H1B	111.0	C12—C13—H13A	123.5
C2—C1—H1B	111.0	S1—C13—H13A	123.5
H1A—C1—H1B	109.0	C19—C14—C15	117.93 (17)
C21—C2—C20	110.38 (17)	C19—C14—C7	122.04 (16)
C21—C2—C3	108.98 (15)	C15—C14—C7	119.98 (15)
C20—C2—C3	111.67 (17)	C16—C15—C14	121.1 (2)
C21—C2—C1	110.00 (15)	C16—C15—H15A	119.5
C20—C2—C1	111.50 (15)	C14—C15—H15A	119.5
C3—C2—C1	104.13 (13)	C17—C16—C15	120.3 (2)
N4—C3—C2	103.18 (14)	C17—C16—H16A	119.8
N4—C3—H3A	111.1	C15—C16—H16A	119.8
C2—C3—H3A	111.1	C16—C17—C18	120.1 (2)
N4—C3—H3B	111.1	C16—C17—H17A	120.0
C2—C3—H3B	111.1	C18—C17—H17A	120.0
H3A—C3—H3B	109.1	C17—C18—C19	120.3 (2)
N4—C5—C6	107.72 (15)	C17—C18—H18A	119.9
N4—C5—H5A	126.1	C19—C18—H18A	119.9
C6—C5—H5A	126.1	C14—C19—C18	120.3 (2)
C5—C6—C7	107.35 (15)	C14—C19—H19A	119.9
C5—C6—C9	123.27 (16)	C18—C19—H19A	119.9
C7—C6—C9	128.43 (15)	C2—C20—H20A	109.5
C8—C7—C6	105.94 (14)	C2—C20—H20B	109.5
C8—C7—C14	123.88 (15)	H20A—C20—H20B	109.5
C6—C7—C14	129.90 (15)	C2—C20—H20C	109.5
N4—C8—C7	108.68 (15)	H20A—C20—H20C	109.5
N4—C8—C1	109.35 (14)	H20B—C20—H20C	109.5
C7—C8—C1	141.97 (15)	C2—C21—H21A	109.5
O1—C9—C6	122.11 (16)	C2—C21—H21B	109.5
O1—C9—C10	119.34 (16)	H21A—C21—H21B	109.5
C6—C9—C10	118.51 (15)	C2—C21—H21C	109.5
C11—C10—C9	130.52 (17)	H21A—C21—H21C	109.5
C11—C10—S1	111.14 (14)	H21B—C21—H21C	109.5
C9—C10—S1	118.32 (13)		
			
C8—C1—C2—C21	−92.43 (17)	C7—C6—C9—O1	23.5 (3)
C8—C1—C2—C20	144.77 (18)	C5—C6—C9—C10	33.8 (3)
C8—C1—C2—C3	24.22 (18)	C7—C6—C9—C10	−158.85 (16)
C5—N4—C3—C2	−165.6 (2)	O1—C9—C10—C11	−160.14 (19)
C8—N4—C3—C2	16.3 (2)	C6—C9—C10—C11	22.1 (3)
C21—C2—C3—N4	92.99 (17)	O1—C9—C10—S1	17.9 (2)
C20—C2—C3—N4	−144.81 (16)	C6—C9—C10—S1	−159.86 (13)
C1—C2—C3—N4	−24.36 (18)	C13—S1—C10—C11	−1.90 (15)
C8—N4—C5—C6	−0.2 (2)	C13—S1—C10—C9	179.73 (15)
C3—N4—C5—C6	−178.29 (19)	C9—C10—C11—C12	179.87 (18)
N4—C5—C6—C7	0.1 (2)	S1—C10—C11—C12	1.8 (2)
N4—C5—C6—C9	169.72 (16)	C10—C11—C12—C13	−0.6 (3)
C5—C6—C7—C8	0.02 (19)	C11—C12—C13—S1	−0.8 (3)
C9—C6—C7—C8	−168.92 (17)	C10—S1—C13—C12	1.57 (19)
C5—C6—C7—C14	−173.97 (16)	C8—C7—C14—C19	−144.22 (17)
C9—C6—C7—C14	17.1 (3)	C6—C7—C14—C19	28.8 (3)
C5—N4—C8—C7	0.2 (2)	C8—C7—C14—C15	33.1 (2)
C3—N4—C8—C7	178.78 (15)	C6—C7—C14—C15	−153.90 (17)
C5—N4—C8—C1	−179.28 (15)	C19—C14—C15—C16	−0.8 (3)
C3—N4—C8—C1	−0.7 (2)	C7—C14—C15—C16	−178.23 (16)
C6—C7—C8—N4	−0.11 (18)	C14—C15—C16—C17	−0.2 (3)
C14—C7—C8—N4	174.33 (14)	C15—C16—C17—C18	1.4 (3)
C6—C7—C8—C1	179.0 (2)	C16—C17—C18—C19	−1.5 (3)
C14—C7—C8—C1	−6.5 (3)	C15—C14—C19—C18	0.7 (3)
C2—C1—C8—N4	−15.16 (18)	C7—C14—C19—C18	178.01 (16)
C2—C1—C8—C7	165.7 (2)	C17—C18—C19—C14	0.5 (3)
C5—C6—C9—O1	−143.84 (19)		

### Hydrogen-bond geometry (Å, º)

**Table:**

*D*—H···*A*	*D*—H	H···*A*	*D*···*A*	*D*—H···*A*
C13—H13*A*···O1^i^	0.93	2.69	3.529 (3)	150

Symmetry code: (i) −*x*+1/2, *y*+1/2, *z*.
